# Supplementary material for: Txnip deletions and missense alleles prolong the survival of cones in a retinitis pigmentosa mouse model
Source: bioRxiv. 2024 Mar 20:2023.08.03.551766. Preprint. [Version 3] doi: 10.1101/2023.08.03.551766 (PMC10871187; doi:10.1101/2023.08.03.551766)
Supplement: Supplement 1 [file NIHPP2023.08.03.551766v3-supplement-1.pdf]

bioRxiv preprint doi: https://doi.org/10.1101/2023.08.03.551766; this version posted March 20, 2024. The copyright holder for this preprint (which was not certified by peer review) is the author/funder, who has granted bioRxiv a license to display the preprint in perpetuity. It is made available under aCC-BY 4.0 International license.

Figure 2-figure supplement 1 Txnip deletions expressed only within RPE cells: quantification of the GLUT1 level within the basal surface of the RPE. GLUT1 expression in P20 wt eyes infected with control (AAV8-RedO-H2BGFP, 2.5 × 10^8 vg/eye), or a Txnip allele (2.5 × 10^8 vg/eye) plus RedO-H2BGFP (2.5 × 10^8 vg/eye), as indicated in each panel. Txnip deletions are detailed in **Figure 4**. Wt Txnip (red group) and Txnip.C247S.LLAA (blue group) were published previously (Xue et al., 2021), and are repeated here using the same conditions as in this study for comparison to the new alleles. Only regions that were infected, as indicated by the H2BGFP marker that was co-injected, were analyzed. GLUT1 intensity from regions of interest (ROIs) within the basal RPE was quantified. Red: GLUT1; green: RedO-H2BGFP for infection tracing; gray: DAPI. Arrows: RPE basal surface. Sample size: control, 26 ROIs from 4 eyes; Txnip.C247S, 9 ROIs from 3 eyes; N.Txnip, 36 ROIs from 6 eyes; sC.Txnip, 10 ROIs from 6 eyes; C.Txnip.CS, 13 ROIs from 3 eyes, C.Txnip.CS.LLAA, 7 ROIs from 3 eyes; nt.Tnip.CS^{320}, 14 ROIs from 4 eyes; wt Txnip, 11 ROIs from 4 eyes; Txnip.CS.LLAA, 21 ROIs from 4 eyes. Error bar: standard deviation. Statistics: Mann-Whitney U Test compared to control with Bonferroni correction. NS: not significant, *** p<0.001, **** p< or <<0.0001.

bioRxiv preprint doi: https://doi.org/10.1101/2023.08.03.551766; this version posted March 20, 2024. The copyright holder for this preprint (which was not certified by peer review) is the author/funder, who has granted bioRxiv a license to display the preprint in perpetuity. It is made available under aCC-BY 4.0 International license.

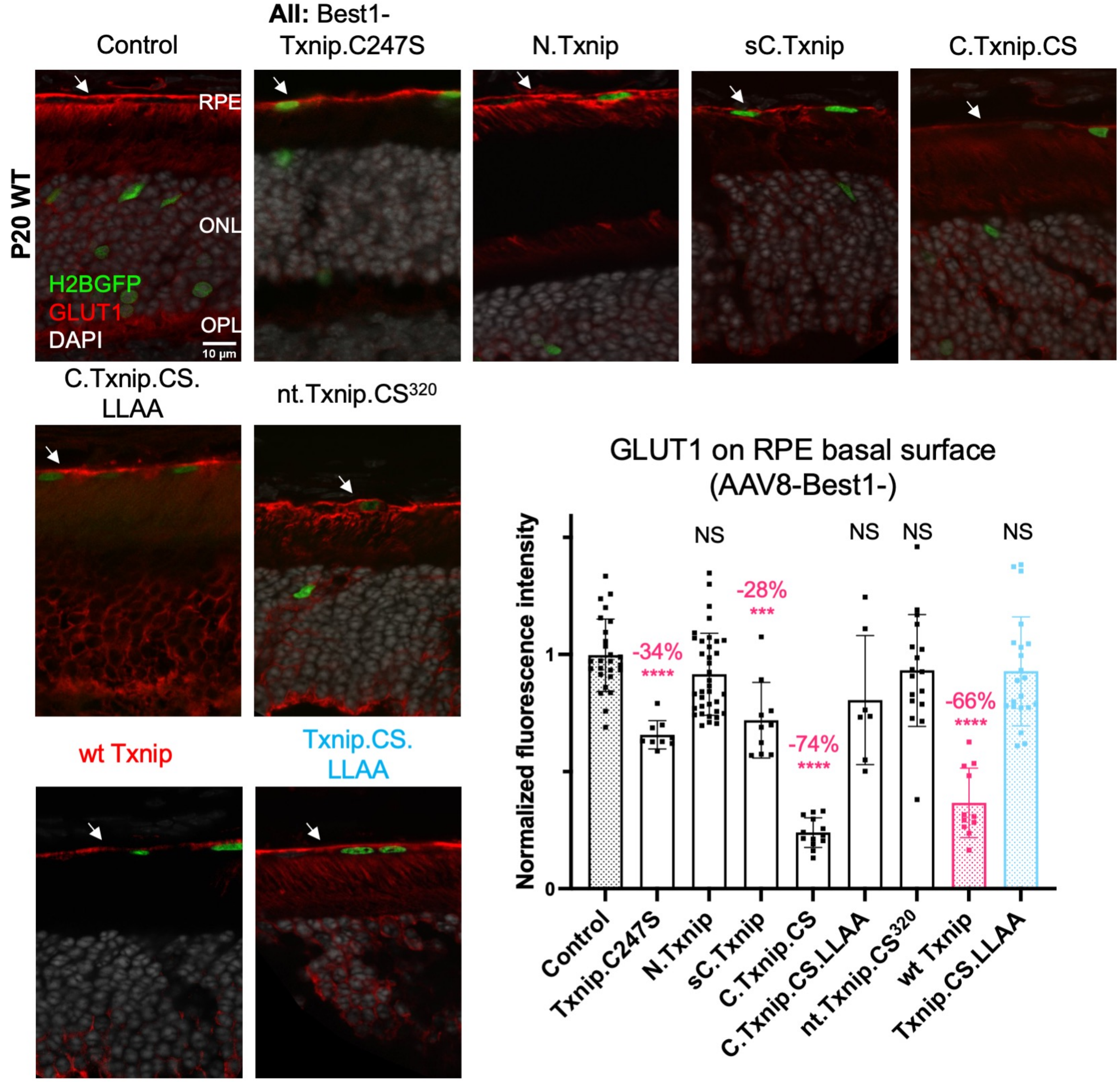

Figure 2 Supplement 1

bioRxiv preprint doi: https://doi.org/10.1101/2023.08.03.551766; this version posted March 20, 2024. The copyright holder for this preprint (which was not certified by peer review) is the author/funder, who has granted bioRxiv a license to display the preprint in perpetuity. It is made available under aCC-BY 4.0 International license.

Figure 2-figure supplement 2 Predicted protein-protein interactions of TXNIP and GLUT1 by an algorithm, ColabFold, based on AlphaFold-2.

bioRxiv preprint doi: https://doi.org/10.1101/2023.08.03.551766; this version posted March 20, 2024. The copyright holder for this preprint (which was not certified by peer review) is the author/funder, who has granted bioRxiv a license to display the preprint in perpetuity. It is made available under aCC-BY 4.0 International license.

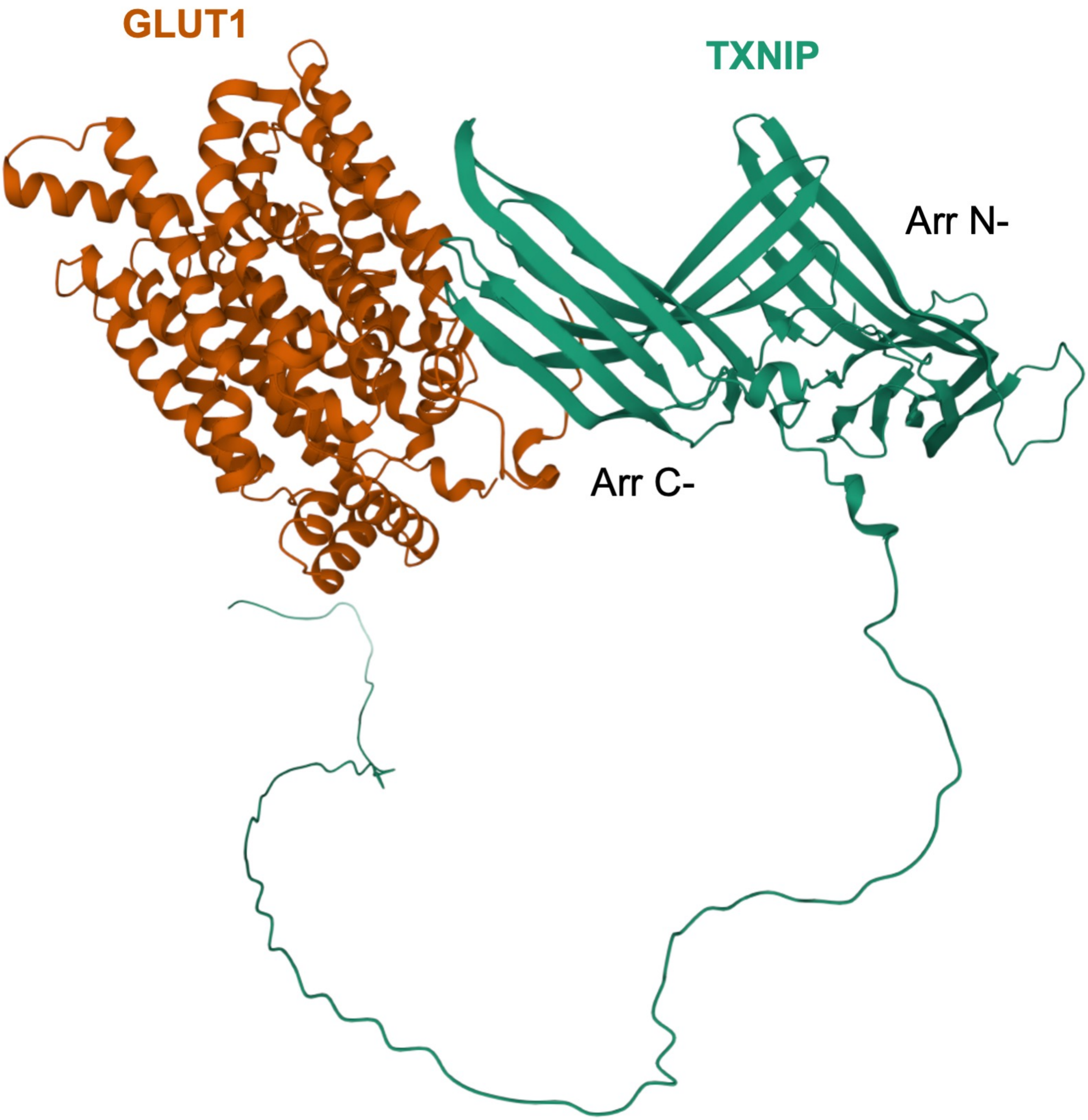

Figure 2 Supplement 2

bioRxiv preprint doi: https://doi.org/10.1101/2023.08.03.551766; this version posted March 20, 2024. The copyright holder for this preprint (which was not certified by peer review) is the author/funder, who has granted bioRxiv a license to display the preprint in perpetuity. It is made available under aCC-BY 4.0 International license.

Figure 5-figure supplement 1 Predicted 3D protein structures of HSP90AB1 and PARP1.

A. Predicted 3D protein structures of HSP90AB1 by AI algorithm AlphaFold-2 from two angles of view.

B. Predicted 3D protein structures of PARP1 by AI algorithm AlphaFold-2 from two angles of view.

bioRxiv preprint doi: https://doi.org/10.1101/2023.08.03.551766; this version posted March 20, 2024. The copyright holder for this preprint (which was not certified by peer review) is the author/funder, who has granted bioRxiv a license to display the preprint in perpetuity. It is made available under aCC-BY 4.0 International license.

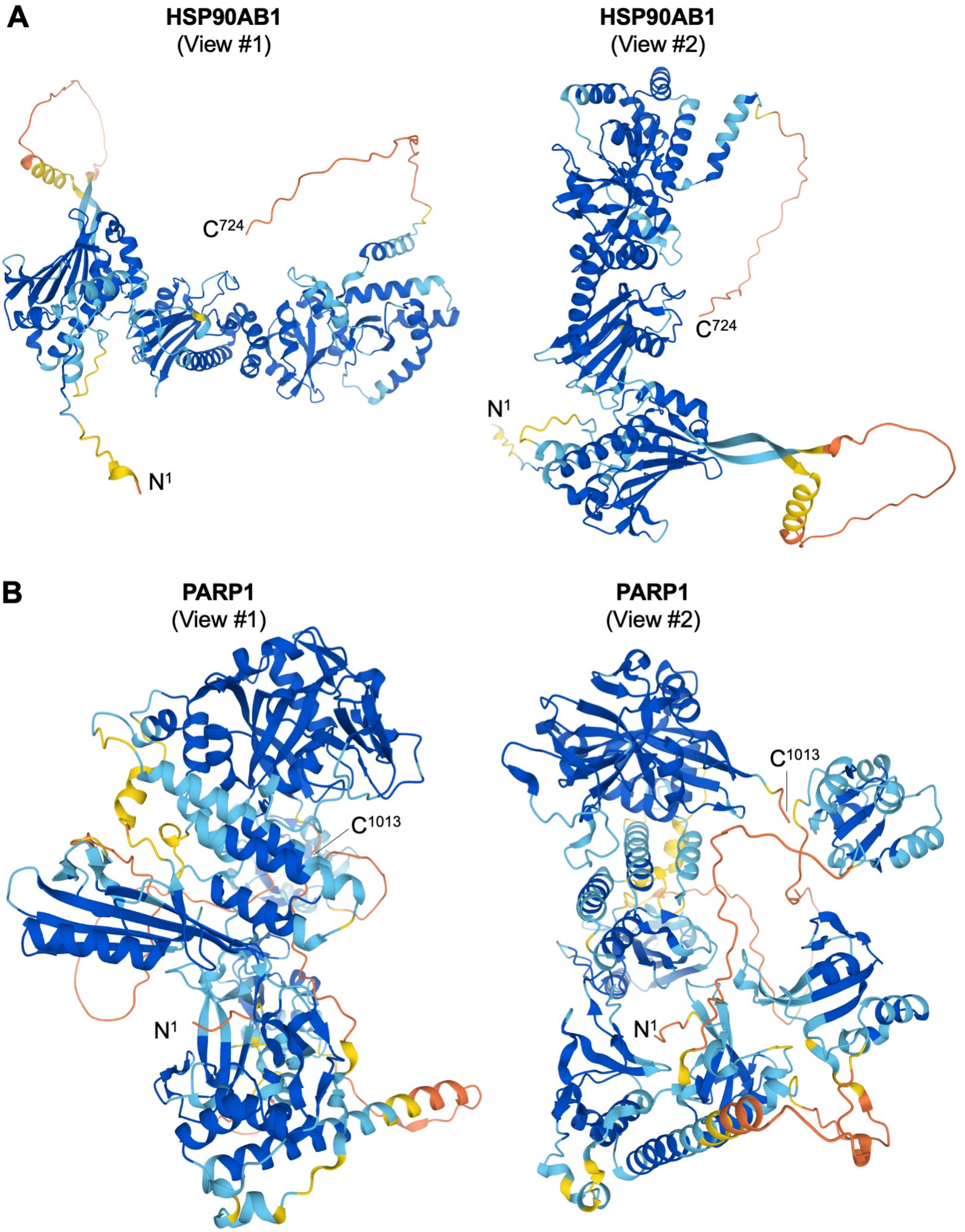

Figure 5 Supplement 1

bioRxiv preprint doi: https://doi.org/10.1101/2023.08.03.551766; this version posted March 20, 2024. The copyright holder for this preprint (which was not certified by peer review) is the author/funder, who has granted bioRxiv a license to display the preprint in perpetuity. It is made available under aCC-BY 4.0 International license.

Figure 5-figure supplement 2 Predicted 3D protein interactions among TXNIP, HSP90AB1, and PARP1 by AI algorithm AlphaFold Multimer from two angles of view. Abbreviations: Arr C-, C-terminal arrestin domain.

bioRxiv preprint doi: https://doi.org/10.1101/2023.08.03.551766; this version posted March 20, 2024. The copyright holder for this preprint (which was not certified by peer review) is the author/funder, who has granted bioRxiv a license to display the preprint in perpetuity. It is made available under aCC-BY 4.0 International license.

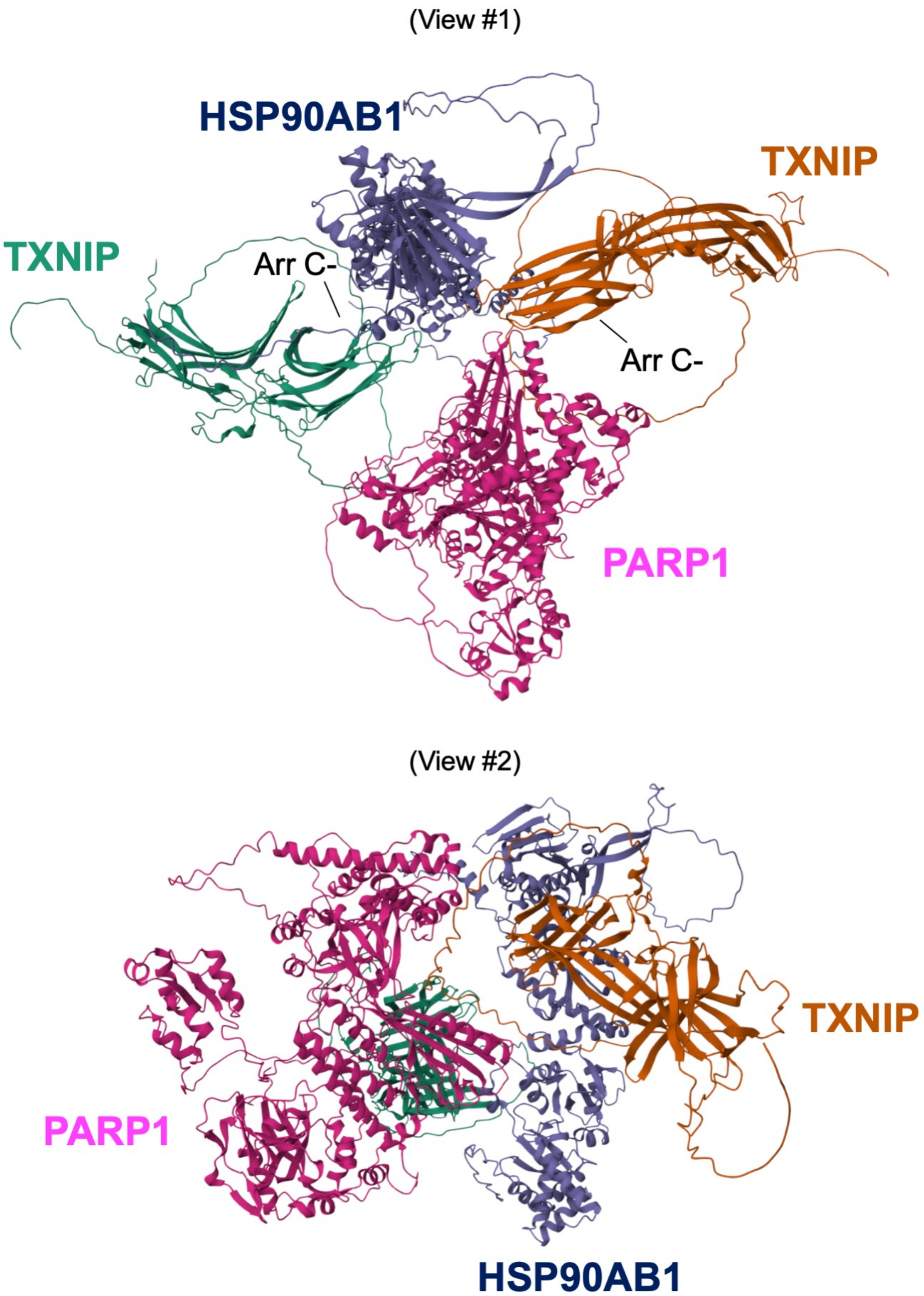

Figure 5 Supplement 2
